# Recent Progress in the Chemical Synthesis of Class II and S-Glycosylated Bacteriocins

**DOI:** 10.3389/fmicb.2018.01048

**Published:** 2018-05-23

**Authors:** François Bédard, Eric Biron

**Affiliations:** ^1^Faculty of Pharmacy and Institute of Nutrition and Functional Foods, Université Laval, Québec, QC, Canada; ^2^Laboratory of Medicinal Chemistry, CHU de Québec Research Centre, Québec, QC, Canada

**Keywords:** bacteriocins, antimicrobial peptides, peptide synthesis, solid-phase peptide synthesis, native chemical ligation, microwave-assisted synthesis

## Abstract

A wide variety of antimicrobial peptides produced by lactic acid bacteria (LAB) have been identified and studied in the last decades. Known as bacteriocins, these ribosomally synthesized peptides inhibit the growth of a wide range of bacterial species through numerous mechanisms and show a great variety of spectrum of activity. With their great potential as antimicrobial additives and alternatives to traditional antibiotics in food preservation and handling, animal production and in veterinary and medical medicine, the demand for bacteriocins is rapidly increasing. Bacteriocins are most often produced by fermentation but, in several cases, the low isolated yields and difficulties associated with their purification seriously limit their use on a large scale. Chemical synthesis has been proposed for their production and recent advances in peptide synthesis methodologies have allowed the preparation of several bacteriocins. Moreover, the significant cost reduction for peptide synthesis reagents and building blocks has made chemical synthesis of bacteriocins more attractive and competitive. From a protein engineering point of view, the chemical approach offers many advantages such as the possibility to rapidly perform amino acid substitution, use unnatural or modified residues, and make backbone and side chain modifications to improve potency, modify the activity spectrum or increase the stability of the targeted bacteriocin. This review summarized synthetic approaches that have been developed and used in recent years to allow the preparation of class IIa bacteriocins and *S*-linked glycopeptides from LAB. Synthetic strategies such as the use of pseudoprolines, backbone protecting groups, microwave irradiations, selective disulfide bridge formation and chemical ligations to prepare class II and *S*-glycosylsated bacteriocins are discussed.

## Introduction

Antimicrobial resistance to antibiotics has become a major challenge in the treatment of infectious diseases, chronic illnesses and immune disorders (Bush et al., 2011; Lewis, 2013; Michael et al., 2014; Ferri et al., 2017). This public health problem is very costly both in care and life and often faces limited treatment options. The unregulated use and misuse of antibiotics in the food and animal production industry as well as veterinary and human medicine led to the emergence of multi-resistant pathogenic strains (Center for Disease Controls and Prevention: U.S. Department of Health and Human Services, 2013; Cotter et al., 2013; Van Boeckel et al., 2015). In this context, new highly effective antimicrobial agents for which resistance cannot be readily acquired are sorely needed in order to maintain the ability of modern medicine to treat bacterial infections. Among the most promising alternatives to conventional antibiotics, bacteriocins show very attractive antimicrobial properties and their potential use as food preservatives, bio-controlling agents or therapeutics has been widely studied in the last decades (Papagianni, 2003; Bali et al., 2016; López-Cuellar et al., 2016; Ahmad et al., 2017; Mathur et al., 2017).

Produced by a wide variety of bacteria to fight other microorganisms in their competitive environments, bacteriocins form a heterogeneous group of peptides with great variations in size, structure and mode of action. Hundreds of these ribosomally synthesized peptides have been identified and characterized over the years and are now described in detail in various databases (Hammami et al., 2007; van Heel et al., 2013). Several approaches have been developed to classify bacteriocins and the classification used in this review is based on the system used to classify the bacteriocins of lactic acid bacteria (LAB) (Cotter et al., 2005, 2013) on their structural characteristics with respect to the nomenclature proposed for ribosomally-synthesized post-translationally modified peptides (RiPPs) (Arnison et al., 2013). Bacteriocins produced by Gram-positive bacteria, such as those from LAB, are divided into three major classes: the heat stable post-translationally modified peptides (class I), the low-molecular weight (<10 kDa) heat stable unmodified peptides (class II) and the heat labile high-molecular-weight proteins (class III). In Gram-negative bacteria, most characterized bacteriocins have been isolated from *Escherichia coli* and other *enterobacteria*, and they are often referred to as microcins (small peptides) or colicins (larger proteins).

The most important appeal of bacteriocins as antibacterial agents and antibiotic substitutes is their multiple advantages over other antimicrobial agents commonly used in food preservation and handling, animal production and in human and veterinary medicine. For example, bacteriocins have been shown to be: (i) safe for consumption since they are completely digested in the gastrointestinal tract (Kheadr et al., 2010; Fernandez et al., 2016), (ii) highly potent (10^3^ to 10^6^ times more than several other antimicrobials including conventional antibiotics); (iii) resistant to common thermal treatments for pasteurization or even sterilization (De Vuyst and Leroy, 2007; Keymanesh et al., 2009; Abriouel et al., 2010). Moreover, several bacteriocins are recognized as GRAS (generally recognized as safe) substances by the United States Food and Drug Administration (FDA) and the European legislation for pharmaceutical and food industry uses. However, despite their great potential and attractive efficacy, the use of bacteriocins remains limited due largely to high production costs usually associated with low production yields and onerous technological requirements. More research and development as well as new approaches are needed in order to make the use of bacteriocins as antimicrobial agents feasible on a larger scale, whether in the food industry or in human health and veterinary medicine.

Chemical synthesis has been proposed for the large-scale production of active bacteriocins. However, very few bacteriocins have been successfully prepared in satisfying yields using such means. Several challenging features that are essential for their bioactivity, such as lasso structure, large macrocycles, and presence of long hydrophobic segments, lanthionines, glycosylated side chains or complicated peptide motifs can make the task very daunting. Beside their production on a large scale, access to bacteriocins by chemical synthesis would allow further molecular engineering to enhance the potency, improve pharmacological properties, increase the stability and modify the spectrum of activity. As a result, new approaches were urgently needed to overcome synthetic pitfalls and efficiently prepare bioactive bacteriocins by chemical synthesis. Fortunately, several developments in peptide synthesis methodologies have been successfully used to prepare several bacteriocins. Because class III bacteriocins are complex large proteins currently inaccessible by chemical approaches and the synthesis of class I lanthibiotic bacteriocins has already been reviewed by Tabor et *al*. (Tabor, 2011), this review will focus on class I *S*-linked glycopeptides and class II bacteriocins produced by Gram-positive bacteria and discuss the recent advances made for their synthesis.

## General Chemical Synthesis Strategies

**FIGURE 1:**
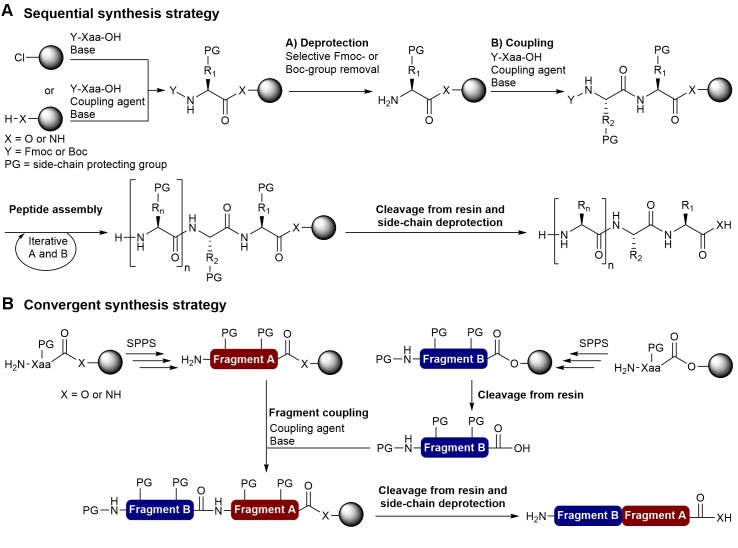
Solid-phase peptide synthesis strategies. **(A)** Stepwise synthesis using the Fmoc/tBu or Boc/Bzl strategy and **(B)** Convergent synthesis.

A wide variety of technologies including chemical synthesis, recombinant DNA technology, cell-free expression systems, transgenic plants or enzymatic synthesis have been developed to produce peptide-based compounds. Generally the choice of the most suitable technology to produce a peptide is based on its size and the chemical synthesis approach is more appropriate and efficient for low-molecular weight peptides (<6 kDa). With the possibility to use unnatural amino acids, introduce pseudo-peptide bonds and perform side chain modifications, chemical synthesis offers access to a more important chemical diversity than peptides produced by recombinant methodologies. With the development of solid-phase peptide synthesis (SPPS), large-scale chemical synthesis has become a viable approach for the production of small- and medium-sized peptides ranging from approximately 5 to 50 residues, and the chemical way is now often a better option than the biotechnological methods of recombinant DNA or biocatalysis for the synthesis of medium-sized peptides. Compared to conventional synthesis in solution where a purification is required after each step, the SPPS approach is considerably more convenient and efficient since the growing peptide chain is attached to an insoluble polymeric support and allows the use of larger amount of reagents to favor reaction completion, simple removal of excess reagents by filtration and washing, and a single purification step once the peptide sequence is completed and removed from the solid support. In the SPPS methodology, *N*^α^-protected amino acids are attached to the N-terminal amine of the growing peptide chain on the solid support followed by deprotection of the amino group (**Figure 1A**). This two-step cycle (coupling and deprotection) is repeated until the peptide sequence is completed and the desired peptide can be obtained after its release from the solid support and removal of the side-chain protecting groups. The chemical synthesis of peptides has remarkably progressed since the first work of Bruce Merrifield on SPPS (Merrifield, 1963). A great number of synthetic improvements including more efficient coupling reagents (Valeur and Bradley, 2009; El-Faham and Albericio, 2011), solid supports (Kates et al., 1998; Rademann et al., 1999; García-Martín et al., 2006; Rapp, 2007), linkers (Boas et al., 2009; Góngora-Benítez et al., 2013), orthogonal protecting groups (Isidro-Llobet et al., 2009), and the use of microwave (MW) irradiations (Collins et al., 2014) have emerged to overcome difficulties associated with SPPS and access a wide variety of peptides (Guan et al., 2015; Behrendt et al., 2016; Paradis-Bas et al., 2016), including bacteriocins.

The two most important SPPS strategies are the linear and convergent synthesis. The linear (sequential) synthesis approach involves the stepwise addition of amino acids until the desired peptide is achieved (**Figure 1A**). Currently, peptides prepared by stepwise amino acid additions via standard SPPS are generally limited to approximately 50 residues (Chen et al., 2015; Guan et al., 2015). Convergent synthesis involves the independent solid-phase synthesis of peptide fragments that are then cleaved from the polymer and linked by condensation on solid support or in solution with standard coupling reagents or chemoselective reactions (chemical ligation) (**Figure 1B**). The convergent approach is often the most appropriate way to synthesize peptides that contain >50 amino acid residues and the development of several chemical ligation methods allowed the preparation of long peptide chains and small proteins (Harmand et al., 2014; Thapa et al., 2014; Chen et al., 2015; Bondalapati et al., 2016). While fragment condensation with standard coupling reagents requires N- or C-terminal and side-chain protected fragments, chemical ligation methods such as the native chemical ligation (NCL) (Dawson et al., 1994), α-ketoacid-hydroxylamine ligation (KAHA) (Bode et al., 2006; Pusterla and Bode, 2015; Bode, 2017), salicylaldehyde (SAL) ester-mediated ligation (Zhang et al., 2013) and traceless-Staudinger ligation (Nilsson et al., 2000; Saxon et al., 2000) are compatible with unprotected peptide fragments.

For both sequential and convergent synthesis approaches, the solid support plays a critical role in peptide assembly and should be mechanically stable, show good swelling properties in commonly used organic solvents, and be compatible with the selected synthetic methodology. The most frequently used solid supports for peptide synthesis are the classic cross-linked polystyrene (PS) resins, polyethylene glycol (PEG)-PS composite resins (e.g., TentalGel^TM^) (Rapp, 2007) and cross-linked PEG resins (e.g., ChemMatrix^®^) (García-Martín et al., 2006). Compared to PS resins, PEG-containing resins are compatible with polar solvents and they were proposed to more suitable for the synthesis of large peptides as they are able to form aggregation-disrupting interactions with growing peptide chains (García-Martín et al., 2006). The Boc/Bzl and Fmoc/tBu synthetic methodologies are the two most common SPPS strategies, and the strategy utilized should be considered when choosing the appropriate type of resin linker (Sewald and Jakubke, 2009; Góngora-Benítez et al., 2013). Several systems for the automated synthesis of peptides covering scales ranging from 1.5 mg to 5 kg and compatible with Boc/Bzl (Boc-SPPS) and Fmoc/tBu SPPS (Fmoc-SPPS) are now available from several companies. Based on the mild acidic conditions for final deprotection and the commercial availability of a wide variety of orthogonally protected amino acids, the Fmoc/tBu approach has been the most commonly used strategy to prepare bacteriocins by chemical synthesis. This review describes the different strategies that have been reported to overcome synthetic pitfalls and access bioactive bacteriocins.

## Synthesis of Class Ii Bacteriocins

Class II bacteriocins are heat stable small peptides containing from 25 to 70 amino acid residues that are largely unmodified, with the exception of disulfide bridges, head-to-tail macrocyclization and N-terminus formylation. LAB are frequently found as producers of class II bacteriocins and members of this class can be further divided into subgroups, including the class IIa, IIb, IIc, and IId covered in this review. Showing variations in size and structure, a great variety of synthetic strategies have been used to overcome pitfalls and successfully prepare members of this class.

### Class IIa Bacteriocins

Also called pediocin-like bacteriocins, members of the class IIa are well-known for their strong antilisterial activity and have been widely studied. Characterized by an N-terminal consensus YGNGV sequence, they contain from 35 to 50 amino acid residues and generally a minimum of two Cys residues involved in a disulfide bond. Several class IIa bacteriocins and analogs thereof have been successfully prepared by chemical synthesis. Total synthesis for leucocin A, pediocin PA-1, sakacin P, curvacin A, mesentericin Y105, enterocin CRL35 and lactococcin MMFII have been reported (**Table 1**). Several studies reported the use of a synthetic class IIa bacteriocin but complete details about their synthesis are unfortunately often missing. The first described total synthesis of class IIa bacteriocins was reported by Fimland et al. (1996) to determine the role of the C-terminal region in bacterial strain specificity. In their study, sakacin P, curvacin A, leucocin A and pediocin PA-1 were prepared by stepwise standard Boc-SPPS. After their release from the resin (not described), the deprotected peptides were obtained with crude purities ranging from 1 to 10%. While sakacin P required two HPLC runs to obtain a 70–80% purity, the purification of pediocin PA-1, curvacin A and leucocin A needed a cation-exchange chromatography and three HPLC runs to reach >80% purity. In this case, the yields obtained were relatively low and found to be about 10% for sakacin P, 3% for curvacin A and leucocin A and about 1% for pediocin PA-1 (**Table 1**).

**Table 1:** Reported synthetic class IIa bacteriocins and methods used for their synthesis.

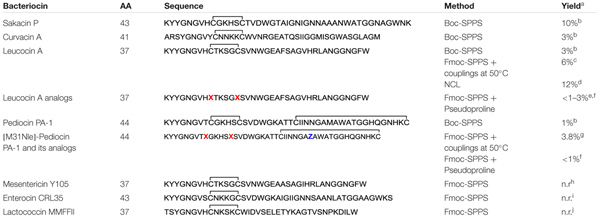


*n.r.: not reported; X = allylglycine, Ser, Phe, norvaline, or Cys; Z = Norleucine (Nle). ^*a*^Overall isolated yield for purified bacteriocins; ^*b*^sequence analysis revealed 85–95% purity for pediocin PA-1 and leucocin A and 70–80% purity for sakacin P and curvacin A (Fimland et al., 1996); ^*c*^leucocin A enantiomer (all-D leucocin A) and amino acids after position 20 were coupled with HATU at 50°C in DMF (Yan et al., 2000); ^*d*^calculated overall yield based on yields obtained for each step (fragment 1 (33%) × fragment 2 (55%) × NCL (98%) × cyclization (70%)) (Bodapati et al., 2013); ^*e*^overall yields based on resin loading (Derksen et al., 2006); ^*f*^overall yields based on resin loading (Derksen et al., 2008); ^*g*^yield for [31Nle]-pediocin PA-1 (Kaur et al., 2004); ^*h*^(Fleury et al., 1996; Castano et al., 2005); ^*i*^(Masias et al., 2017); ^*j*^(Ferchichi et al., 2001).*

**FIGURE 2:**
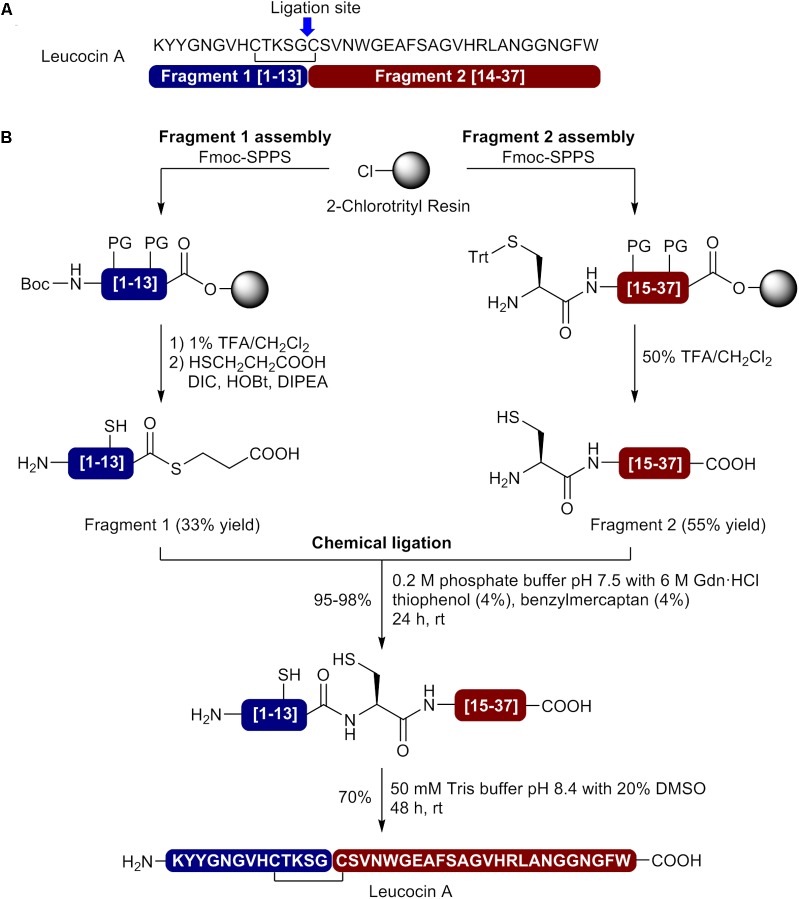
Synthesis of leucocin A using native chemical ligation. **(A)** Leucocin A sequence showing the selected ligation site and identification of the fragments. **(B)** Assembly of the N- and C-terminal fragments by Fmoc-SPPS, native chemical ligation and oxidative cyclization (Bodapati et al., 2013).

In order to overcome synthetic pitfalls and increase the yields, researchers have introduced several SPPS strategies, including the use of pseudoprolines, specialized resins, heating during coupling and NCL. The case of leucocin A, a bacteriocin of 37nostoc gelidium, is particularly interesting since several strategies have been used to achieved higher yields and develop bioactive analogs (**Table 1**). To produce leucocin A by standard Fmoc-SPPS, Fleury et al. (1996) used a polyamide/kiesel guhr resin bearing the 4-hydroxymethylphenoxyacetic acid linker (HMPA) and were able to isolated the bacteriocin in 16% crude yield before purification. In another study, Yan et al. prepared the enantiomer of leucocin A (composed of D-amino acids) by stepwise Fmoc-SPPS with D-amino acids on Wang resin using the coupling reagent 2-(1H-benzotriazole-1-yl)-1,1,3,3-tetramethyluronium hexafluorophosphate (HBTU) for the 20 first residues and 2-(7-aza-1H-benzotriazole-1-yl)-1,1,3,3-tetramethyluronium hexafluorophosphate (HATU) at 50°C for the remaining positions (Yan et al., 2000). This simple modification in the synthetic pathway allowed the synthesis of all-D leucocin A in a 6% overall yield after cleavage from the resin and purification by HPLC (**Table 1**). In other reports from the same group, several unnatural analogs of leucocin A, such as [9,14]-dicarba-, [9,14]-diallyl-, [C9S, C14S]-, and [C9F, C14F]-leucocin A analogs were prepared on NovaSyn TGA resin (HMPA-PEG-PS) by incorporating pseudoproline dipeptides at positions 14/15 [Cys-Ser(Ψ^Me,Me^Pro)] and/or 22/23 [Phe-Ser(Ψ^Me,Me^Pro)] in the sequence to minimize on-resin aggregation of the growing peptide chain (Derksen et al., 2006; Derksen et al., 2008). Unfortunately, low overall yields have been obtained for these leucocin A analogs (**Table 1**). More recently, Bodapati et al. (2013) used a convergent NCL strategy to prepare bioactive leucocin A (**Figure 2**). In their approach, the Cys at position 14 was selected as the ligation site (**Figure 2A**) and the two fragments have been prepared by standard Fmoc-SPPS on 2-chlorotrityl resin. After its release from the resin, the protected N-terminal fragment (fragment 1 [1–13]) was submitted to thioesterification with ethyl 3-mercaptopropionate in presence of *N*,*N*′-diisopropylcarbodiimide (DIC), *N*-hydroxybenzotriazole (HOBt) and *N*,*N*-diisopropylethylamine (DIPEA) and precipitated in cold diethyl ether. The protecting groups were removed under acidic conditions in presence of trifluoroacetic acid (TFA) and the fragment 1 thioester obtained in 33% yield after purification by HPLC. On the other hand, the C-terminal fragment (fragment 2 [14–37]) was isolated in 55% yield after cleavage from the resin, side chain deprotection and purification by HPLC. Next, the two fragments were coupled by NCL in phosphate buffer (pH 7.6) containing 6 M guanidinium chloride (GdnHCl) and thiophenol (4%)/benzyl mecaptan (4%) as catalysts (**Figure 2B**). Monitoring of the reaction showed that the ligation was completed in 24 h. The ligation product was purified by HPLC and isolated in 98% yield. Finally, the disulfide bond was formed by air oxidation in Tris buffer (pH 8.4) containing DMSO (20%) for 48 h and the cyclic product subjected to HPLC purification to afford pure leucocin A in 70% yield. The overall yield for the synthesis of leucocin A using the NCL approach was 12% [fragment 1 (33%) × fragment 2 (55%) × NCL (98%) × cyclization (70%)] (**Table 1**).

Pediocin PA-1, a bacteriocin of 44 amino acid residues from *Pediococcus acidilactici* containing two disulfide bonds between Cys9-Cys14 and Cys24-Cys44, is another very interesting case since the problems associated with its production by recombinant technologies and chemical synthesis continue to limit its applicability and delay regulatory approval. The very high sensitivity of the methionine at position 31 to aerobic oxidation is the first challenge for the production of this bacteriocin. Pediocin PA-1 oxidized at this position is 100 times less active (Fimland et al., 1996) and no currently used methods for disulfide bond formation have been able to prevent Met31 oxidation. To avoid this problem, Kaur et al. (2004) replaced the methionine at position 31 by norleucine. The peptide was prepared by stepwise standard Fmoc-SPPS on Wang resin and by coupling the amino acids with HBTU in DMF for the first 15 residues and HATU at 50°C in *N*-methylpyrrolidone (NMP) for the remaining positions. After cleavage from the resin and side chain deprotection with a TFA cocktail, the linear precursor was purified by HPLC. The Acm protecting groups on Cys9 and Cys14 were removed along with disulfide bond formation with I_2_ in MeOH (0.1 M) for 2 h followed by quenching with aqueous ascorbic acid (1 M). A final HPLC purification afforded [M31Nle]-pediocin PA-1 in 3.8% overall yield (**Table 1**). Antimicrobial activity assays showed that the synthetic [M31Nle]-pediocin PA-1 was equally potent as the natural pediocin PA-1 against *Listeria innocua* and *Carnobacterium divergens* (Kaur et al., 2004). In another study from the same group, several [M31Nle]-pediocin PA-1 analogs were prepared on 2-chlorotrityl resin by incorporating pseudoproline dipeptides at positions 21/22 and 34/35 [Ala-The(Ψ^Me,Me^Pro)] in each case and 14/15 [Phe-Ser(Ψ^Me,Me^Pro)] for [9,14]-diPhe and 7/8 [Val-Thr(Ψ^Me,Me^Pro)] for [9,14]-diallyl analogs (Derksen et al., 2008). Unfortunately, after their release from the resin, side chain deprotection and purification by HPLC, the [M31Nle]-pediocin PA-1 analogs have been isolated in <1% overall yields, based on resin loading (**Table 1**).

While several reports on the synthesis of leucocin A and pediocin PA-1 and analogs thereof have been published, detailed total syntheses for other class IIa bacteriocins are substantially rarer. The synthesis of mesentericin Y105, a class IIa bacteriocin from *Leuconostoc mesenteroides* containing 37 amino acids, was achieved by standard Fmoc-SPPS on HMPA-PEG-PS resin using DIC or HBTU as coupling reagent (Fleury et al., 1996; Castano et al., 2005). Similarly, a bacteriocin of 43 amino acids from *Enterococcus mundtii* CRL35 and named enterocin CRL35 has been prepared on Rink amide resin using standard Fmoc-SPPS (Saavedra et al., 2004; Emilse et al., 2015; Masias et al., 2017). Other class IIa bacteriocins such as the lactococcin MMFII from *Lactococcus lactis* (Ferchichi et al., 2001) have been synthesized but detailed information about their preparation and yield are not reported in the published study. Carnobacteriocin B2 from *Carnobacterium piscicola* has been prepared with its leader sequence in <1% overall yield (Sprules et al., 2004).

### Class IIb Bacteriocins

Class IIb bacteriocins, also known as two-peptide bacteriocins, act by the complementary action of two different peptides. Compared to pediocin-like bacteriocins, members of the class IIb have been less studied and reports on their synthesis are rarer. Among the few reported studies on class IIb bacteriocins, we were able to find only one that uses a synthetic bacteriocin. In this case, the complementary peptides lactocin 705α (GMSGYIQGIPDFLKGYLHGISAANKHKKGRLGY) and lactocin 705β (GFWGGLGYIAGRVGAAYGHAQASANNHHSPING) from *Lactobacillus casei* CRL705 were prepared by standard Fmoc-SPPS (Cuozzo et al., 2000; Castellano et al., 2007). Unfortunately, the peptides were prepared by an external company and information about their preparation and isolated yields could not be found.

### Class IIc Bacteriocins

Class IIc bacteriocins are head-to-tail cyclic bacteriocins containing from 35 to 70 amino acids. The exceptionally large size of the macrocycle and the high content of hydrophobic residues found in several members of this class pose serious synthetic challenges for their preparation. Several macrocyclization methodologies, including a thia-zip cyclization/desulfurization combination have been attempted for the final ring closure but none were able to yield the final circular bacteriocin.

**FIGURE 3:**
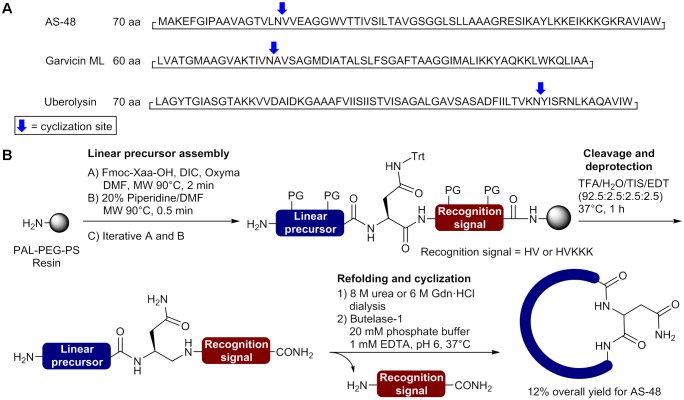
Synthesis of circular bacteriocins using the butelase chemoenzymatic approach. **(A)** Sequences and selected macrocyclization sites. **(B)** Preparation of the linear precursor and butelase-mediated macrocyclization (Hemu et al., 2016).

The first successful total synthesis of circular bacteriocins has been achieved by Hemu et al. (2016) using a chemoenzymatic approach. In this breakthrough study, the enzyme butelase I, an Asp/Asn specific ligase isolated from the leguminous plant *Clitoria ternatea*, was used to perform the final head-to-tail macrocyclization (Nguyen et al., 2014, 2016). Butelase I recognizes the tripeptide NHV motif in a precursor and eliminates the HV dipeptide in the ligated product (Cao et al., 2015; Nguyen et al., 2015, 2016). It also displays a broad specificity for the incoming sequence with no preference for the N-terminal P1 position and favoring hydrophobic residues (Leu, Val, Ile) at the P2 position (Nguyen et al., 2014, 2016). The most attractive advantages of the reported butelase-mediated macrocyclization are the absence of extra sequence in the ligation product (traceless reaction) and the possibility to add polar amino acids in the recognition signal to increase aqueous solubility of the linear precursor. The only unavoidable requirement is the presence of an Asn or Asp residue in the peptide sequence. In the described study, the butelase-mediated macrocyclization was applied to prepare the circular bacteriocins AS-48, uberolysin and garvicin ML, each containing at least one Asn (**Figure 3A**). Linear precursors containing a C-terminal NHV recognition signal were first assembled on PEG-PS resin bearing the 5-(3,5-dimethoxy-4-(aminomethyl)phenoxy)pentanoic acid (PAL) linker by stepwise MW-assisted Fmoc-SPPS in only 6 h using DIC and ethyl 2-cyano-2-(hydroxyimino)acetate (Oxyma) as coupling reagents (**Figure 3B**) (Collins et al., 2014). A supplementary C-terminal KKK sequence was added on AS-48 and uberolysin linear precursors to increase aqueous solubility of highly hydrophobic segments found in their sequence and facilitate butelase-mediated cyclization. After their release from the resin with a TFA cocktail, the linear precursors were purified by HPLC. Direct macrocyclization of the unfolded linear precursors by the traceless butelase-mediated ligation was unsuccessful. To overcome this setback and possibly brings the N and C termini in close proximity, the linear precursors were refolded by dissolution in 8 M urea or 6 M GdnHCl at 50–100 mM followed by dialysis for folding (**Figure 3B**). To perform the butelase-mediated macrocyclization, Hemu et *al*. used a peptide concentration of 50 μM in a sodium phosphate buffer (20 mM Na_2_HPO_4_, 1 mM EDTA, pH 6) with a peptide: enzyme ratio of 100:1 and observed that the ring closing reaction at 37°C was completed in 1 h for AS-48, 0.5 h for garvicin ML and 24 h for uberolysin with yields of 85%, 90 and 93%, respectively. To accelerate the procedure, the authors performed a one-pot synthesis of AS-48 where the purification of the linear precursor was bypass. With only a final purification required, the entire process for the synthesis of AS-48 was completed in less than 24 h (6 h for the linear precursor synthesis, 5 h for refolding, 1 h for the macrocyclization and 1 h for the purification) and the circular bacteriocin obtained with an excellent 12% overall yield. This chemoenzymatic approach developed by Tam and coworkers is really promising and will certainly be applied for the synthesis of unusually large cyclic peptides and circular proteins. Unfortunately butelase is not readily available and is produced by extraction from pods of *Clitoria ternatea* (Nguyen et al., 2014), as it cannot be produced by recombinant technologies at the present time. The development of new methodologies to produce recombinant ligases will certainly promote the use of enzyme-mediated ligation and cyclization in the synthesis of bacteriocins, complex peptides and proteins (Yang et al., 2017).

**FIGURE 4:**
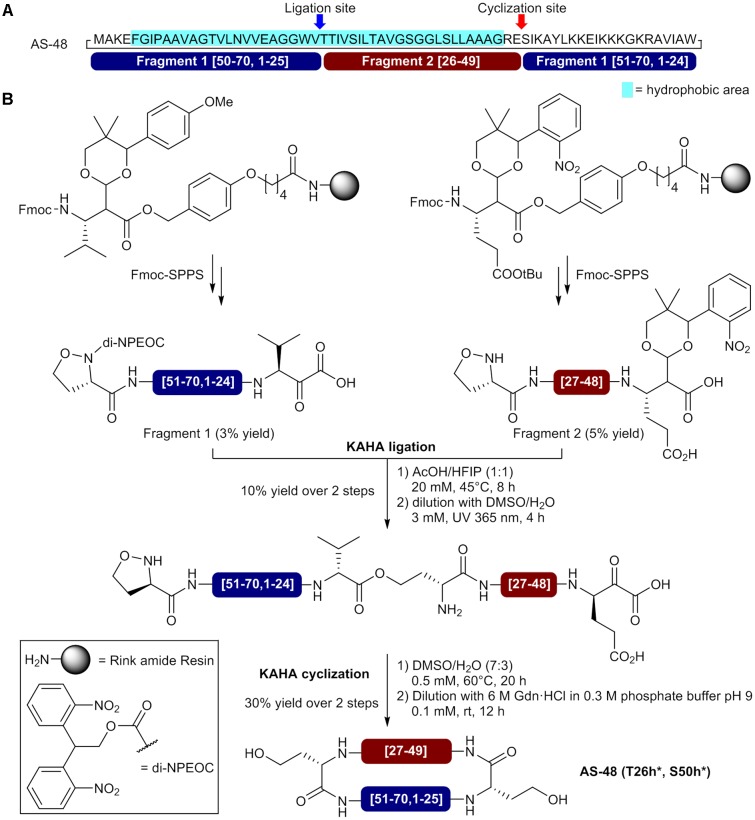
Synthesis of a homoserine-mutant of the circular bacteriocin AS-48 by sequential KAHA ligation and macrocyclization. **(A)** Sequence of AS-48 showing the selected ligation and macrocyclization sites and composition of the fragments. **(B)** Preparation of the N- and C-terminal fragments and circular bacteriocin synthesis by KAHA ligation and macrocyclization (Rohrbacher et al., 2017). h^∗^ = homoserine.

More recently, Rohrbacher et al. (2017) reported the preparation of a homoserine-mutant of AS-48 by using a convergent synthesis with α-ketoacid-hydroxylamine (KAHA) ligations for linear precursor assembly and final macrocyclization. Compatible with organic and aqueous solvents, the KAHA ligation relies on the chemoselective reaction of ketoacids with hydroxylamines to generate an amide bond and is well-suited for assembling hydrophobic peptides and proteins (Harmand et al., 2014; Rohrbacher et al., 2015; Bode, 2017). In the reported approach, the authors use two fragments bearing an *N*-terminal (*S*)-5-oxaproline as cyclic hydroxylamine to generate homoserine residues at the ligation and macrocyclization sites after reaction with C-terminal α-ketoacids and *O*- to *N*-acyl transfers (**Figure 4A**). An important property of the KAHA approach is the formation of depsipeptide bonds (ester bonds) as primary ligation products leaving free polar amino groups at the reaction site (**Figure 4B**). This interesting feature can facilitate the assembly of hydrophobic sequences before the *O*- to *N*-acyl transfer and final folding. For the synthesis of the circular bacteriocin AS-48, the selection of V25-T26 as the ligation site and E49-S50 as the cyclization sites was based on a suitable hydrophobicity distribution for each fragment and the lowest impact of homoserine residues on the bioactivity (**Figure 4A**). Moreover, in order to minimize handling and avoid tedious purification of the hydrophobic linear precursor, the authors used photolabile protecting groups for the C-terminal α-ketoacid of fragment 1 and N-terminal 5-oxaproline of fragment 2. A valine α-ketoacid for fragment 1 and a photoprotected glutamic acid α-ketoacid for fragment 2 were attached to Rink amide resins via a Wang-type linker and both fragments were assembled by standard Fmoc-SPPS using *O*-(6-chlorobenzotriazol-1-yl)-*N*,*N*,*N*′,*N*′-tetramethyluronium hexafluorophosphate (HCTU) and HATU as coupling reagents (**Figure 4B**) (Thuaud et al., 2016). Following cleavage from the resin and side chain deprotection with a TFA cocktail, the fragments were purified by HPLC and obtained in 3–5% yields. Fragments 1 and 2 were then subjected to KAHA ligation in a mixture of acetic acid (AcOH) and hexafluoroisopropanol (HFIP) (1:1) at 45°C and the reaction was completed in 8 h. After N- and C-termini deprotection under UV irradiations at 365 nm, the linear precursor was purified by HPLC and obtained in 10% yield. The KAHA macrocyclization was performed with the linear precursor in aqueous DMSO (0.5 mM) at 60°C and completed in 20 h. Finally, the mixture was diluted with 0.3 M phosphate buffer containing 6 M GdnHCl to induce the *O*- to *N*- acyl shifts. After 12 h, both rearrangements were completed and HPLC purification afforded pure homoserine mutant AS-48 (T26h^∗^, S50h^∗^) in 30% yield for the entire macrocyclization process. Surprisingly, the synthetic cyclic bacteriocin showed lower antibacterial activity (MIC >10 mM) than the reported value of 0.5 μM for the natural peptide. While incomplete folding was observed in circular dichroism spectra, the authors showed that the synthetic circular bacteriocin was able to adopt correct folding and recover full activity after storage for 1 month at 4°C in buffer at pH 3. Using the KAHA ligation for fragments condensation and macrocyclization, the authors were able to obtain 1 mg of bioactive homoserine-mutant AS-48 in 0.005% overall yield, based on the loading of preloaded resins. Despite the low yield, the results obtained by Rohrbacher et *al*. demonstrated the utility of the KAHA ligation for the synthesis of hydrophobic proteins via the formation of depsipeptide bonds and its compatibility with acidic conditions in the presence of organic solvents such as AcOH/HFIP that can efficiently dissolve difficult sequences. The KAHA ligation approach is really promising and will certainly be used again for the synthesis of unusually large cyclic peptides and circular proteins.

### Class IId Bacteriocins

Finally, the class IId includes linear non-pediocin-like one-peptide bacteriocins. As observed with class IIb bacteriocins, very few syntheses for members of the class IId have been described in details. Among the few reported studies using synthetic class IId bacteriocins, the 43 amino acids durancin A5-11a (MGAIAKLVTKFGWPLIKKFYKQIMQFIGQGWTIDQIEKWLKRH) and the 44 amino acids durancin A5-11b (MGAIAKLVAKFGWPIVKKYYKQIMQFIGEGWAINKIIEWIKKHI) from*Enterococcus durans* A5-11 have been successfully prepared using MW-assisted Fmoc-SPPS on Rink amide resin (Belguesmia et al., 2013). In another study, the 22 amino acids bactofancin A (KRKKHRCRVYNNGMPTGMYRWC) has also been synthesized by MW-assisted Fmoc-SPPS but using the cross-linked PEG resin ChemMatrix^®^ bearing the 4-(4-hydroxymethyl-3-methoxyphenoxy)-butyric acid (HMPB) linker (O’Shea et al., 2013; Guinane et al., 2016). Unfortunately detailed information about their preparation and isolated yields are not reported in the published studies. The use of synthetic epidermicin NI01 (Gibreel and Upton, 2013), a *N*-terminal formylated bacteriocin from *Staphylococcus epidermis* 224 containing 51 amino acid residues (*N*-formyl-MAAFMKLIQFLATKGQKYVSLAWKHKGTILKWINAGQSFEWIYKQIKKLWA), has also been reported but the peptide was prepared by an external company and information about its synthesis could not be found (Sandiford and Upton, 2012).

## Synthesis of S-Glycosylated Bacteriocins

Glycocins are class I bacteriocins containing at least one carbohydrate moiety linked to a Cys residue via its side chain. In some reports, they can also be found in the class IV as complex bacteriocins containing a carbohydrate moiety. These *S*-linked glycopeptides are rare and only three *S*-glycosylated bacteriocins have been isolated and characterized until now; namely, glycocin F (Stepper et al., 2011), sublancin 168 (Wang and van der Donk, 2011) and thurandacins A and B (Wang et al., 2014). While the *in vitro* reconstitution of the biosynthesis of thurandacins A and B using recombinant technologies has been reported (Wang et al., 2014), glycocin F and sublancin 168 were recently successfully prepared by chemical synthesis.

Sublancin 168 is a glycocin of 37 amino acids produced by *Bacillus subtilis* 168 containing a β–*S*-linked glucose moiety on the Cys22 residue and two disulfide bonds between Cys7-Cys36 and Cys14-Cys29 (Oman et al., 2011; Wang and van der Donk, 2011). The first total synthesis of sublancin 168 was achieved by Katayama et al. (2011) using a thioglycosylamino acid unit (Fmoc-Cys(Glc(OAc)_4_)-OH) in stepwise Fmoc-SPPS (Katayama et al., 2011). The linear precursor was first assembled on Wang resin by coupling the amino acids at 50°C with*N*,*N*′-dicyclohexylcarbodiimide (DCC) and HOBt in NMP for 1 h. Moreover, a pseudoproline dipeptide (Gly-The(Ψ^Me,Me^Pro)) was incorporated at positions 18/19 during peptide elongation. After cleavage from the resin and side chain deprotection with a TFA cocktail, a first disulfide bond was formed between Cys7 and Cys37 in a solution of 6 M urea/5% hydrazine/10% DMSO. The monocyclic product was purified by HPLC and isolated in 5% yield. The Acm protecting groups on Cys14 and Cys29 were removed along with disulfide bond formation using I_2_ in MeOH (20 mM) for 1 h at 40°C followed by quenching with aqueous ascorbic acid (1 M). A final HPLC purification afforded sublancin 168 in 20% yield for the cyclization step. By using a stepwise synthesis strategy, the authors were able to produce sublancin 168 with a 1% overall yield based on resin loading.

**FIGURE 5:**
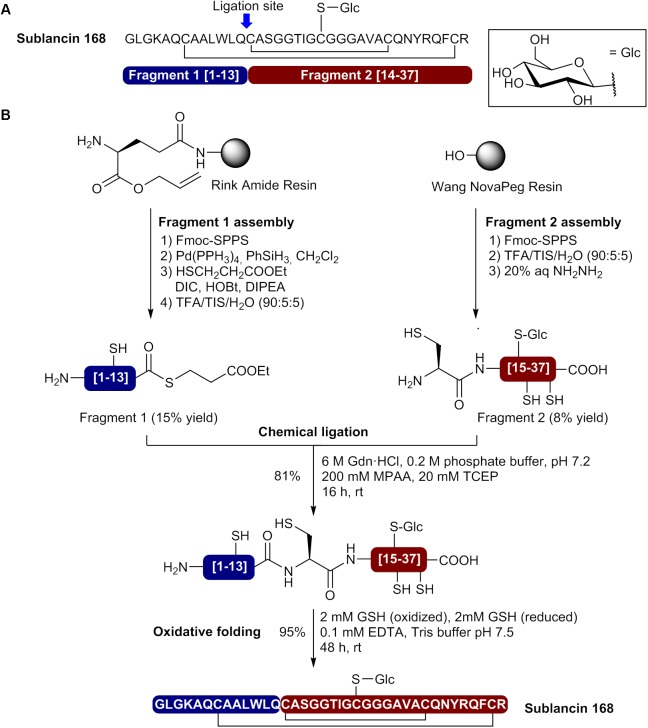
Synthesis of sublancin 168 by a convergent native chemical ligation approach. **(A)** Sequence of sublancin 168 showing the selected ligation site and composition of the fragments. **(B)** Preparation of the N- and C-terminal fragments and bacteriocin synthesis by native chemical ligation and oxidative folding (Hsieh et al., 2012).

More recently, Hsieh et al. used a convergent NCL approach to prepare sublancin 168 and *S*-glycosylated analogs (Hsieh et al., 2012). In their approach, the Cys at position 14 was selected as the ligation site and two segments have been prepared by MW-assisted Fmoc-SPPS (**Figure 5A**). The N-terminal fragment (fragment 1 [1–13]) was assembled on Rink amide resin bearing a side chain anchored Fmoc-Glu-Oallyl (**Figure 5B**). This side chain anchoring strategy allowed on-resin selective demasking of the C-terminal carboxylate with Pd(PPh_3_)_4_ and thioesterification using ethyl-3-mercaptopropionate, DIC, HOBt and DIPEA as base. The peptide thioester was released from the resin and the side chains deprotected by treatment with a TFA cocktail. Fragment 1 was obtained in 15% yield after purification by HPLC. The C-terminal fragment (fragment 2 [14–37]) was prepared on Wang NovaPEG resin and the beforehand prepared thioglycosylamino acid Fmoc-Cys(Glc(OAc)_4_)-OH introduced at position 22 during peptide synthesis (**Figure 5B**). Moreover, 2,4-dimethoxybenzyl (Dmb) derived dipeptides Fmoc-Gly-(Dmb)Gly-OH were incorporated at positions 17/18 and 23/24 to prevent aggregation during peptide elongation. After cleavage from the resin and side chain deprotection by acidolysis, the *O*-acetate protecting groups on the D-glucose moiety were removed with aqueous hydrazine. Purification of the resulting product afforded fragment 2 in 8% yield. Next, the two segments were subjected to NCL in presence of mercaptophenylacetic acid (MPAA) as thiol catalyst and tris(2-carboxyethyl)phosphine (TCEP) as reductant in 6 M GdnHCl/phosphate buffer at pH 7.2 (**Figure 5B**). The reaction was completed after 16 h and the ligated product isolated in 81% yield after HPLC purification. Finally, the linear precursor was cyclized using oxidized and reduced glutathione (GSH) and purification by HPLC afforded sublancin 168 in 95% yield. In this case, the use of a convergent NCL approach allowed the production of sublancin 168 with a 1% overall yield.

**FIGURE 6:**
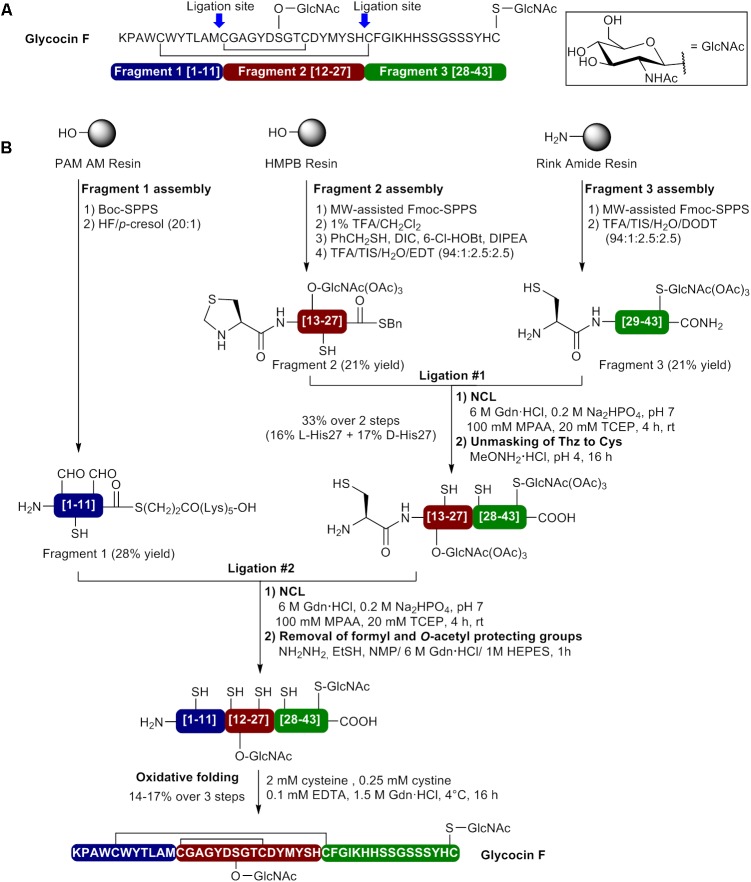
Synthesis of glycocin F by a convergent native chemical ligation approach. **(A)** Sequence of glycocin F showing the selected ligation sites and composition of the fragments. **(B)** Preparation of the three fragments and bacteriocin synthesis by sequential native chemical ligation and oxidative folding (Brimble et al., 2015).

Glocycin F, a 43 amino acid glycopeptide from *Lactobacillus plantarum* KW30, contains two β–linked *N*-acetylglucosamine (GlcNAc) moieties and two disulfide bonds between Cys5-Cys28 and Cys12-Cys21 (**Figure 6A**) (Stepper et al., 2011; Venugopal et al., 2011). While one GlcNAc unit is attached to the sulfur atom of Cys43, the other is linked to side chain oxygen of Ser18. Both GlcNAc moieties have been shown to be essential for glycocin F activity. The first total synthesis of glycocin F was recently reported by Brimble et al. (2015) using a convergent NCL approach. This case is very interesting since several synthetic strategies such as Boc- and Fmoc-SPPS, MW irradiations, incorporation of a pseudoproline dipeptide and N-terminal thiazolidine (Thz) for Cys masking have been combined to successfully achieve the synthesis of bioactive glycocin F. In the described approach, ligation sites at positions 12 and 28 have been selected and involved the use of three fragments to perform two ligation steps and reach the full length peptide (**Figure 6**). First, the side chain glycosylated amino acids Fmoc-Cys(GlcNAc(OAc)_3_)-OH and Fmoc-Ser(GlcNAc(OAc)_3_)-OH were beforehand prepared in solution and introduced in their respective segments during peptide elongation by SPPS. The C-terminal fragment (fragment 3 [28–43]) was assembled on Rink amide resin using MW-assisted Fmoc-SPPS and the *S*-glycosylated amino acid Fmoc-Cys(GlcNAc(OAc)_3_)-OH introduced as the first residue (**Figure 6B**). A pseudoproline dipeptide [Ser(tBu)-Ser(Ψ^Me,Me^Pro)] was incorporated at positions 35/36 during peptide elongation. Fragment 3 (H-Cys28-Cys43(GlcNAc(OAc)_3_)-NH_2_) was obtained in 21% yield after resin cleavage and protecting groups removal with a TFA cocktail containing 2,2′-(ethylenedioxy)diethane thiol (DODT) and HPLC purification. The central fragment (fragment 2 [12–27]) was prepared on PS resin bearing the acid labile HMPB linker by MW-assisted Fmoc-SPPS and the *O*-glycosylated amino acid Fmoc-Ser(GlcNAc(OAc)_3_)-OH introduced position 18 during peptide elongation (**Figure 6B**). The fragment has been capped with an N-terminal thiazolidine residue as a latent Cys to allow the second NCL step. The fully protected peptide fragment was release from the resin using 1% TFA in CH_2_Cl_2_ and the C-terminal carboxylate thioesterified using benzyl mercaptan in presence of DIC, 6-Cl-HOBt and DIPEA as base. Treatment of the protected peptide thioester with a TFA cocktail containing ethylene dithiol (EDT) and purification by HPLC afforded segment 2 thioester (Thz12- Ser18(GlcNAc(OAc)_3_)His27-SBn) in 21% yield. Fragments 2 and 3 were then coupled under NCL conditions in phosphate buffer containing 6 M GdnHCl, MPAA and TCEP at pH 7 and the reaction was completed in 4 h to afford the first ligation product Thz12-Ser18(GlcNAc(OAc)_3_)- Cys43(GlcNAc(OAc)_3_)-NH_2_. At this point, LC-MS analyses showed the presence of two ligation products in a 1:1 ratio having identical mass spectra. As NCL has been shown to proceed with stereochemical integrity (Lu et al., 1996; Kent, 2009; Thapa et al., 2014), the authors hypothesized that complete racemization of the His27 on the C-terminus of segment 2 took place during the thioesterification step with benzyl mercaptan in presence of DIC, 6-Cl-HOBt and DIPEA (Jones et al., 1980). After the unmasking of Thz12 to Cys12 using methoxyamine⋅HCl at pH4, the diastereomers were successfully separated during HPLC purification and obtained in 16.6 and 17.5% yield. As the L-His27 and D-His27 epimers could not be unambiguously assigned, both diastereomers were used in the next steps. The N-terminal fragment (fragment 1 [1–11]) bearing a C-terminal S(CH_2_)_2_CO-(Lys)_5_-OH thioester tail was assembled on 4-(hydroxymethyl)phenylacetamidomethyl resin (PAM AM resin) using standard Boc-SPPS. After cleavage from the resin with anhydrous HF/p-cresol (20:1), the fragment H-Lys1-Met11-COS(CH_2_)_2_CO-(Lys)_5_-OH containing *N*_in_-formyl-protected Trp4 and Trp6 was purified by HPLC and isolated in 28% yield. Next, the ligation products were separately subjected to NCL with fragment 1 using the same conditions as the first ligation and the reaction was completed in 4 h. Removal of the formyl and *O*-acetyl protecting groups on the separated epimeric products was achieved with a mixture of hydrazine and 2-mercaptoethanol in NMP/GdnHCl/HEPES to give the epimeric linear precursors H-Lys1-Ser18(GlcNAc(OAc)_3_)-Cys43(GlcNAc(OAc)_3_)-NH_2_. Oxidative folding was performed with a redox couple containing 2 mM cysteine, 0.25 mM cystine and 0.1 mM EDTA in 1.5 M GdnHCl (pH 8.2) at 4°C for 16 h and HPLC purification afforded glycocin F epimers in 14 and 17% yield over three steps. The HPLC profiles of the synthetic glycocin F epimers were compared to the natural product and the second epimer was identified as the one containing L-His27 since it showed the same retention time as the natural Glycocin F. Furthermore, the second epimer was 3 times more active than the synthetic D-His27 glycocin F. By using a convergent NCL approach, the authors were able to produce glycocin F with a <1% overall yield.

## Conclusion and Future Perspectives

There has been considerable progress over recent years in peptide synthesis and previously inaccessible bacteriocins such as *S*-glycopeptides and circular bacteriocins can now be prepared by chemical synthesis. The syntheses described herein show that the methodologies and approaches that have been used until now to prepare bacteriocins are very diverse. A wide variety of solid supports, linkers, coupling reagents and solvents have been involved in the chemical synthesis of bacteriocins and the Fmoc-SPPS approach has emerged as the most frequently used strategy. While the synthesis of some small bacteriocins was performed straightforwardly by standard stepwise SPPS, other bacteriocins could not be accessed by conventional peptide synthesis methodologies. The most frequently encountered synthetic challenge during the preparation of bacteriocins is certainly the presence of hydrophobic segments that can lead to peptide self-aggregation during elongation and hinder the coupling of residues toward the N-terminus. Fortunately, several strategies have been developed to prevent or disrupt self-aggregation and overcome this synthetic pitfall. Among them, PEG-based resins, turn-inducing residues like pseudoprolines and backbone protecting groups, special solvent mixtures and couplings at higher temperature with conventional heating or microwave irradiations have been used in several syntheses described herein for the preparation of bacteriocins. Gaining a lot of popularity in peptide synthesis, the use of higher temperature and microwave-assisted synthesis have emerged as very efficient methods to significantly reduce the required time for peptide assembly, perform difficult couplings and achieve long peptide sequences. The convergent approach using chemical ligations has also experienced tremendous progress in recent years and will certainly allow the synthesis of several other and larger bacteriocins in the coming years. At present time, the most important setback to the production of bacteriocins by chemical synthesis on an industrial scale is the low obtained yields. It is important to note that the syntheses described herein were not developed for large-scale production but to obtain pure bacteriocins and their analogs in sufficient quantity for antimicrobial assays, structural determination by NMR and mechanistic studies. Optimization of the different steps involved in their synthesis would undoubtedly allow the production of bacteriocins with better yields and on a large-scale. The most important appeal of chemical synthesis to produce bacteriocins is the possibility to easily perform molecular engineering to enhance the potency, improve pharmacological properties, increase the stability and even generate chimeras of different classes. Complementary to recombinant technologies, the chemical synthesis approach will certainly help fulfill the needs for bioactive bacteriocins and analogs thereof in the food industry, animal production and veterinary and human medicine.

## Author Contributions

All authors listed have made a substantial, direct and intellectual contribution to the work, and approved it for publication.

## Conflict of Interest Statement

The authors declare that the research was conducted in the absence of any commercial or financial relationships that could be construed as a potential conflict of interest.
